# Repetitive vascular occlusion stimulus (RVOS) versus standard care to prevent muscle wasting in critically ill patients (ROSProx):a study protocol for a pilot randomised controlled trial

**DOI:** 10.1186/s13063-019-3547-5

**Published:** 2019-07-24

**Authors:** Ismita Chhetri, Julie E. A. Hunt, Jeewaka R. Mendis, Stephen D. Patterson, Zudin A. Puthucheary, Hugh E. Montgomery, Benedict C. Creagh-Brown

**Affiliations:** 1 Intensive Care Unit, Royal Surrey County Hospital NHS Foundation Trust, Guildford, GU2 7XX UK; 2 Faculty of Health and Medical Sciences, School of Biosciences and Medicine, University of Surrey, Guildford, UK; 3 Faculty of Sport, Health and Applied Science, St Mary’s University, London, UK; 4 William Harvey Research Institute, Barts and The London School of Medicine and Dentistry, Queen Mary University of London, London, UK; 5 Institute for Sport, Exercise and Health, University College London, London, UK; 6 Department of Medicine, Centre for Human Health and Performance, University College London, London, UK; 7 Intensive Care Unit, Royal Free London NHS Foundation Trust, London, UK; 8 Centre for Human and Applied Physiological Sciences, King’s College London, London,, UK

**Keywords:** Repetitive vascular occlusion stimulus, ICU-acquired weakness, Blood flow restriction, Critical illness, Rehabilitation, Muscle atrophy, Vascular dysfunction

## Abstract

**Background:**

Forty per cent of critically ill patients are affected by intensive care unit-acquired weakness (ICU-AW), to which skeletal muscle wasting makes a substantial contribution. This can impair outcomes in hospital, and can cause long-term physical disability after hospital discharge. No effective mitigating strategies have yet been identified.

Application of a repetitive vascular occlusion stimulus (RVOS) a limb pressure cuff inducing brief repeated cycles of ischaemia and reperfusion, can limit disuse muscle atrophy in both healthy controls and bed-bound patients recovering from knee surgery. We wish to determine whether RVOS might be effective in mitigating against muscle wasting in the ICU. Given that RVOS can also improve vascular function in healthy controls, we also wish to assess such effects in the critically ill. We here describe a pilot study to assess whether RVOS application is safe, tolerable, feasible and acceptable for ICU patients.

**Methods:**

This is a randomised interventional feasibility trial. Thirty-two ventilated adult ICU patients with multiorgan failure will be recruited within 48 h of admission and randomised to either the intervention arm or the control arm. Intervention participants will receive RVOS twice daily (except only once on day 1) for up to 10 days or until ICU discharge.

Serious adverse events and tolerability (pain score) will be recorded; feasibility of trial procedures will be assessed against pre-specified criteria and acceptability by semi-structured interview. Together with vascular function, muscle mass and quality will be assessed using ultrasound and measures of physical function at baseline, on days 6 and 11 of study enrolment, and at ICU and hospital discharge. Blood and urine biomarkers of muscle metabolism, vascular function, inflammation and DNA damage/repair mechanism will also be analysed. The Health questionnaire will be completed 3 months after hospital discharge.

**Discussion:**

If this study demonstrates feasibility, the derived data will be used to inform the design (and sample size) of an appropriately-powered prospective trial to clarify whether RVOS can help preserve muscle mass/improve vascular function in critically ill patients.

**Trial registration:**

ISRCTN Registry, ISRCTN44340629. Registered on 26 October 2017.

**Electronic supplementary material:**

The online version of this article (10.1186/s13063-019-3547-5) contains supplementary material, which is available to authorized users.

## Background

Increasing numbers of patients are being admitted to intensive care units (ICUs) and mortality rates have fallen [1–3]. However, survival comes at the expense of increased dependency and debility: 50% of patients of working age do not return to work; 70% require assistance with daily living activities in the year following discharge; and physical disability can persist for many years [4, 5]. The UK’s National Institute of Health and Care Excellence (NICE) has declared such post-ICU debility to be a public health issue [6].

Impaired quality of life and functional limitation partly result from ICU-acquired weakness (ICU-AW) [7–10]. This affects approximately 40% of adult ICU patients [11], with long-term ventilated or severely septic patients being most commonly and severely affected [12–16]. ICU-AW is associated with poorer outcomes: prolonged mechanical ventilation, increased ICU and hospital length of stay, increased hospital and post-discharge mortality rates, chronic functional disability and reduced quality of life among survivors [8, 17–20]. As a consequence, hospital health-care costs are 30% higher in those affected, with further excess costs relating to the need for rehabilitation, frequent re-admissions and social care upon discharge [18].

The pathophysiology of ICU-AW is multifactorial [10, 21–23]. Whilst neuropathies may occur [24], skeletal muscle wasting plays a central role [25]. The cross-sectional area of the rectus femoris (thigh) muscle (RFCSA) falls by an average of 18% in just 10 days in ventilated ICU patients, and to the greatest degree in those with the largest burden of organ failure [21]. This is underpinned by impaired skeletal muscle protein synthesis and, simultaneously, increased protein breakdown [21, 26], to which altered muscle metabolism [27] and disuse may contribute [23, 28]. The putative role of drugs such as corticosteroids, neuromuscular junction blocking agents and aminoglycoside antibiotics is contested [16, 29–31].

To date, no therapies exist for prevention or treatment of ICU-AW [32, 33]. Early mobilisation and physical activity regimes have proven ineffective [34–38], perhaps because muscle wasting has already occurred. There is thus growing interest in the early application of non-volitional interventions such as neuromuscular electrical stimulation, although proof of benefit remains lacking [38–42].

Application of a repetitive vascular occlusion stimulus (RVOS) shows promise as a mitigating intervention. Repeated inflation/deflation of a blood pressure cuff around a limb to above arterial pressure (~ 200 mmHg) elicits brief bouts (~ 5 min) of limb ischaemia/reperfusion [43]. In healthy subjects, RVOS can improve exercise performance [44–47], whilst vascular occlusion during low-intensity exercise (blood flow restriction exercise) can enhance hypertrophic and strength responses in skeletal muscle [48] of healthy controls [49–52], athletes [53] and the elderly [54–60], and seems to improve physical function and health-related quality of life in patients with inflammatory muscle disease [61–63]. Moreover, RVOS may mitigate against atrophy induced by immobilisation and unloading: patients recovering from ligament reconstruction surgery who received two sessions of RVOS (five cycles of vascular occlusion for 5 min and release for 3 min) to the proximal thigh daily for > 10 days after surgery had 50% less disuse knee extensor muscles mass loss [43]. Similarly, in healthy volunteers with experimentally induced limb immobilisation, RVOS alone or in combination with exercise preserved muscle strength and mass [64, 65].

The mechanism by which RVOS prevents muscle atrophy remains unclear. Application of RVOS enhanced a murine mTOR signalling pathway involved in protein synthesis [66], whilst increased skeletal muscle oxidative capacity is also implicated [67–70].

In addition to impacts on muscle mass, RVOS improves local and systemic endothelial function and microcirculation in healthy controls [71, 72], perhaps through increases in plasma concentration of vascular endothelial growth factor (VEGF) and endothelial progenitor cells [73]. However, it is unknown whether RVOS can prevent or offset macrovascular and microvascular dysfunction observed in bed-rest/ immobilised individuals [74–76] and critically ill patients [77–79].

Of note, such benefits are seen locally (i.e. in the muscle distal to the occlusion), but also *remotely*—in tissue distant from the site of occlusion (so-called ‘remote preconditioning’). RVOS provides protection against myocardial and renal tissue ischaemic injury at the time of vascular intervention or cardiac surgery [80–85]. Unilateral daily arm RVOS over a week improves bilateral vascular function [71], while a longer (300 consecutive days), more frequent (twice daily), protocol improves cerebral perfusion and reduces recurrent strokes by 30% in patients with intracranial arterial stenosis [86]. The mechanism of such remote benefit has yet to be described [87].

Given its remote effects, application of RVOS to a single limb could potentially prevent muscle wasting and vascular dysfunction at both local and remote sites in critically ill patients, thus mitigating against the development of ICU-AW. Evidence suggests that this approach would be safe, with no reports of elevation of muscle damage markers, such as creatinine kinase and myoglobin, and oxidative stress markers, such as lipid peroxide, during blood flow restriction exercise [50, 52, 88, 89]. Moreover, RVOS pre or post exercise seems to be protective against exercise-induced muscle damage [90, 91]. Amongst 12,642 recipients (including healthy subjects, older subjects and individuals with clinical conditions), attributable serious side effects were few: venous thrombus (0.055%), pulmonary embolism (0.008%) and rhabdomyolysis (0.008%) [92]. No negative impact on haemodynamic, haemostatic and inflammatory responses has been observed in healthy young, older and clinical populations [61, 93–95]. In addition to being safe, RVOS also appears well tolerated, with a pain score of 3.6 ± 3.4 (mean ± SD) out of 10 during application to the lower limb in patients with aneurysmal subarachnoid haemorrhage [96].

We thus wish to determine whether the application of RVOS might help maintain muscle mass and improve vascular function in ICU patients. We here describe a trial designed to assess the feasibility of performing such an appropriately-powered study.

## Methods

### Primary objectives

The primary objective of the trial is to determine whether it is safe and tolerable to apply RVOS to a proximal lower limb of ICU patients.

### Secondary objectives

Our secondary objectives are to determine the feasibility of screening; obtaining consent and recruiting; randomising; retaining patients; delivering the trial intervention; performing outcome assessments and collecting data; assessing acceptability for patients, personal consultees (next of kin) and staff; and to determine outcome data characteristics to design a future larger trial.

In addition, we will explore any impacts on measures of muscle mass, quality and function (strength and physical function measures); vascular function; blood and urine biomarkers of muscle metabolism; vascular function; inflammation and DNA damage/repair mechanisms; and clinical outcomes to inform powering of any future prospective trial.

### Study design

**Fig. 1:**
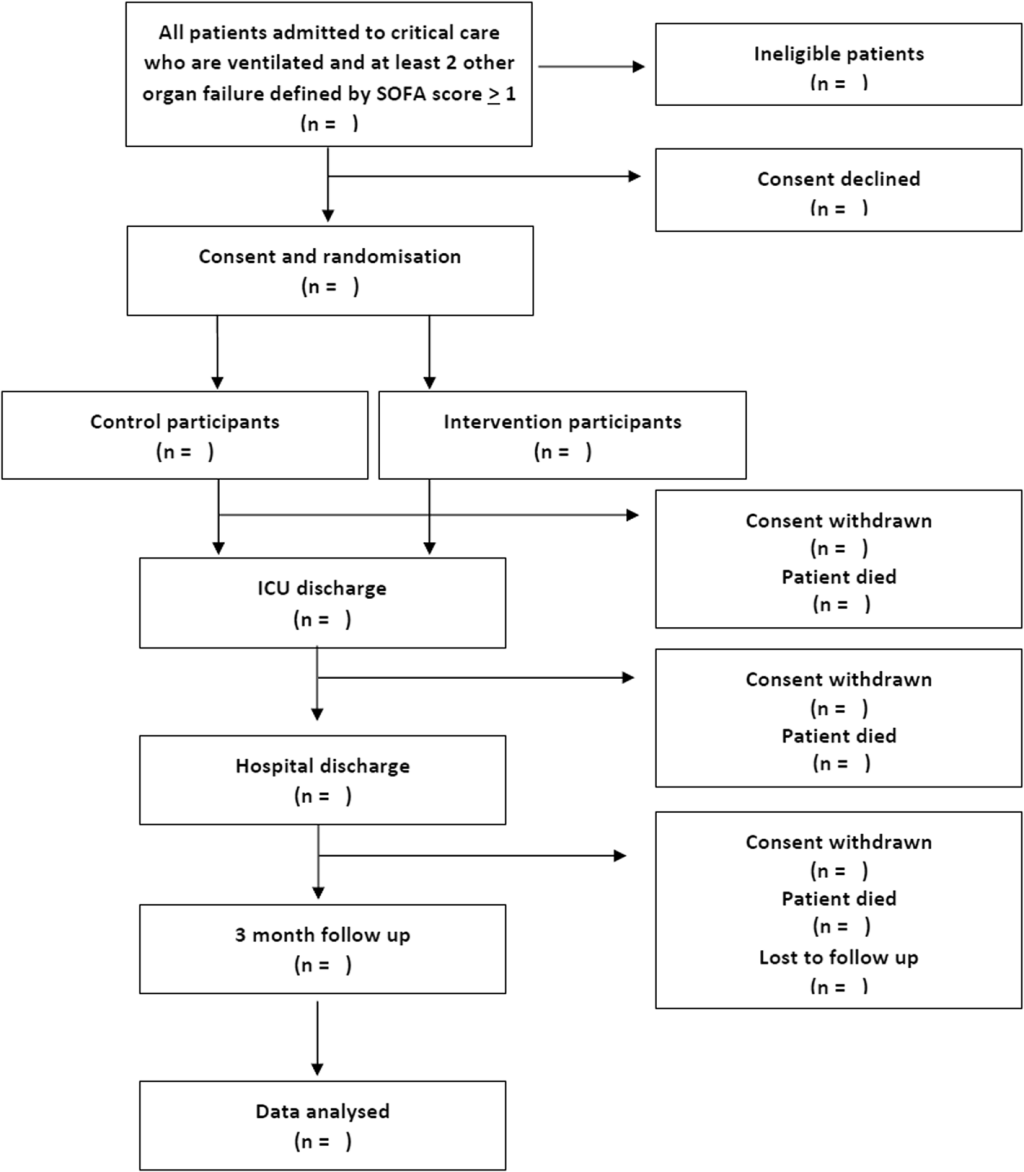
Study flow diagram. ICU intensive care unit, SOFA Sequential Organ Failure Assessment

ROSProx is a partially blinded interventional feasibility trial with randomisation. Eligible patients will be randomised to an intervention arm or a control arm. Patient randomised to the control arm will receive standard care, while intervention patients will receive RVOS treatment in addition to all other standard care. The study protocol has been developed in accordance with the Standard Protocol Items: Recommendations for Interventional Trials (SPIRIT) guidelines (see Additional file 1 for the SPIRIT checklist). The study flow diagram is shown in Fig. 1.

### Study setting

The study will take place within the ICUs of two hospitals in England: Royal Surrey County Hospital NHS Trust (RSCH); and Ashford and St Peter’s Hospital (ASPH) NHS Trust.

### Sample size rationale

A total of 30 participants is recommended for pilot and feasibility trials [97]. To allow for balance in the stratification factors (gender and study site), a sample size of 32 participants was chosen, which will allow us to estimate a failure to deliver RVOS in the intervention arm of 30% to within a 95% confidence interval of ± 15.9%.

Based on an anticipated average rate of recruitment of 6 patients/month, 50% screening failure due to weekends, 20% decline of consent and 30% death before trial completion, we aim to recruit 1.6 patients per site per month. Recruiting from two sites, one starting 2 months after the other, is expected to yield 47 patients over 16 months. Recruitment will continue until 32 ICU survivors have been studied (16 patients in each group).

### Study population

Patients that meet the eligibility criteria (Table 1) will be recruited from each participating ICU over a 16-month period.

**Table 1:** Inclusion and exclusion criteria

Inclusion criteria	Exclusion criteria
1. Age ≥ 18 years 2. Patient admitted to the ICU within the past 48 h 3. Personal consultee provides declaration of agreement for patient enrolment, retrospective patient consent 4. Non-invasive ventilation (CPAP) or invasive mechanical ventilation 5. At least two other organ failures as defined by scoring ≥ 1 points on two of the SOFA score domains 6. Likely to remain in the ICU for at least 4 days	1. Profound cardiovascular instability—infused vasopressors ≥ 0.5 μg/kg/min of norepinephrine; or in opinion of senior attending doctor 2. Profound coagulopathy (prothrombin time > 2.5 times normal, APTT > 2 times normal or platelet count ≤ 50), bleeding diathesis or on intravenous heparin infusion APTR ≥ 2 3. Neuromuscular condition—any previous or concurrent neurological condition or muscle disease 4. History of peripheral arterial vascular disease—any previous surgery or interventional procedure for peripheral arterial insufficiency; or any reason to clinically suspect arterial insufficiency of the leg, such as collateral history of claudication or examination findings of absent peripheral pulses 5. Prior amputation of a lower limb 6. Thigh circumference > 77 cm (technical limitations) 7. Unlikely to survive the ICU 8. Disseminated malignancy 9. Pregnancy 10. Previous, or current, deep vein thrombosis and/or pulmonary embolism 11. Positioned prone 12. Contraindication to pharmacological venous thromboembolism prophylaxis 13. Pre-existing significant cognitive impairment 14. Enrolled in a conflicting interventional trial 15. Lack of ability to communicate in verbal and written English 16. Patient hospitalised > 48 h prior to ICU admission 17. Frail skin, skin condition or soft tissue infection or other reason that prevents experimental use of upper limb

*APTR* activated partial thromboplastin time ratio, *APTT* activated partial thromboplastin time, *CPAP* continuous positive airway pressure, *ICU* intensive care unit, *SOFA* Sequential Organ Failure Assessment

### Recruitment and randomisation process

All sequential ICU admissions will be screened for eligibility. Eligible patients with mental capacity for informed consent will be approached, the risks and benefits of participation explained and written informed consent will be obtained before enrolment by the investigator physician. However, we envision that the majority of eligible patients will be receiving invasive mechanical ventilation and requiring sedation, and thus lacking capacity to consent. In this instance, declaration of agreement will be sought from the patient’s ‘Personal Consultee’ who may be a representative, partner or close friend. Once the participant recovers and is capable of understanding the details of the trial, they will be approached to provide their informed consent retrospectively. If the patient chooses to withdraw from the trial, they will be given the choice of having their existing data and samples destroyed or excluded from final analysis.

Upon informed consent or declaration of agreement, eligible patients will be randomised to either the intervention arm or the control arm in a 1:1 ratio. Separate randomisation lists have been prepared for each site by an independent statistician and uploaded to the study electronic data capture system PROMASYS (Surrey Clinical Trials Unit). In addition, the randomisation list and corresponding envelopes containing randomisation assignments will be stored in the trial file. Randomisation, outcome measure assessments and RVOS intervention will be performed by a research assistant unblinded to patient allocation.

### Study protocol

**Fig. 2:**
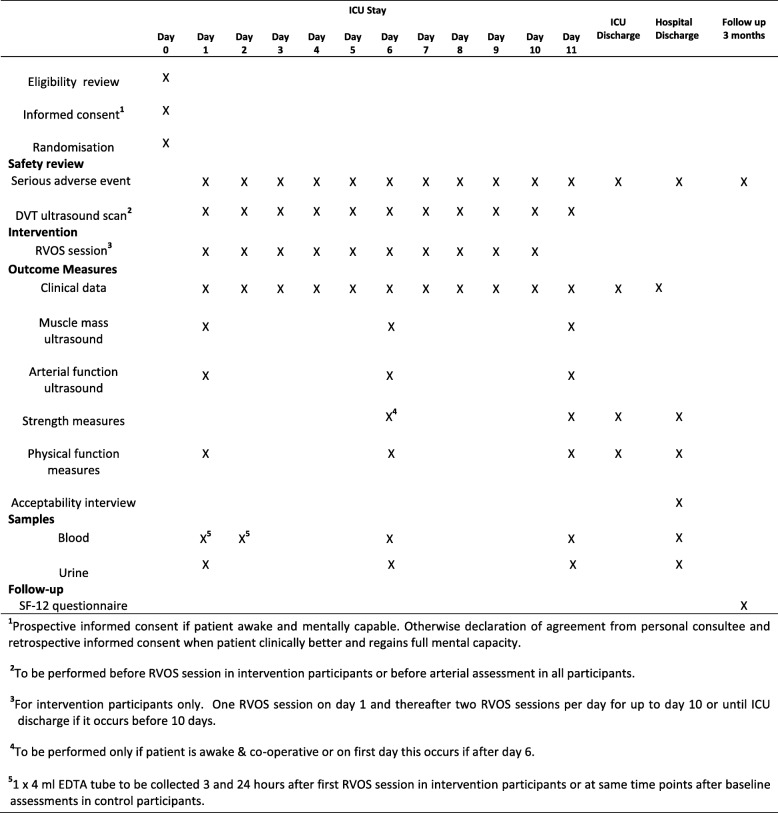
Schedule of study procedures and outcome measure assessments. DVT deep vein thrombosis, EDTA ethylenediamine tetraacetic acid, ICU intensive care unit, RVOS repetitive vascular occlusion stimulus, SF-12 12-Item Short Form Health Questionnaire

Figure 2 illustrates the schedule of study procedures and outcome measure assessments. Briefly, within 24 h of enrolment and randomisation, baseline assessments will be completed. These will include ultrasound assessment of muscle and arterial vascular function; blood and urine sample collection and physical function assessment using the ICU mobility score; recording of clinical characteristics such as medical history, diagnosis and severity of illness (assessed using the Sequential Organ Failure Assessment (SOFA) score); documentation of physical function (assessed using the Katz Index of Independence in Activities of Daily Living score) and nutritional status (assessed using the Malnutrition Screening Tool) prior to illness.

If the participant is randomised to the intervention, one RVOS session will be carried out after baseline assessment on day 1. Arterial blood samples will be collected 3 and 24 h after RVOS and at the same time points after baseline assessments in control participants. From day 2, intervention participants will receive two RVOS sessions per day until day 10 of enrolment or until ICU discharge if this occurs before day 10.

Outcome measure assessments of muscle mass and quality, arterial function, muscle strength, physical function, and blood and urine biomarkers will be repeated on days 6 and 11 of study enrolment. Clinical data including blood results, nutritional and organ support, severity of illness, fluid balance, physiotherapy interventions and specific drug treatment (neuromuscular blocking drugs, corticosteroids, statins and propofol) will be recorded daily from days 1 to 11 of enrolment. At ICU and hospital discharge, physical function and muscle strength assessments will be repeated. In addition, at hospital discharge, an acceptability interview will be conducted with the participant and personal consultee, and blood and urine samples collected. Study participation will end upon completion (by telephone) of the SF-12 health questionnaire 3 months after hospital discharge.

### Repetitive vascular occlusion stimulus (RVOS) intervention

Participants in the intervention arm will receive one session of RVOS treatment on day 1 and two sessions per day, at least 4 h apart, from day 2 to 10 of study enrolment or until ICU discharge if this occurs before day 10. For safety, the intervention leg will be examined daily before the first RVOS session for deep vein thrombosis (DVT) from the common femoral to popliteal vein using ultrasound (CX50 Philips Ultrasound with L12–3 linear array transducer). If DVT is suspected, RVOS or arterial assessment will not be performed and the clinical team will be notified immediately.

**Fig. 3:**
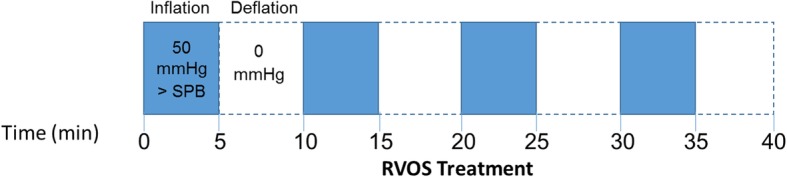
Protocol for repetitive vascular occlusive stimulus (RVOS). Each session of RVOS includes four repetitions of 5-min inflation of pneumatic cuff to 50 mmHg supra-systolic blood pressure (SBP) followed by 5 min of complete (0 mmHg) cuff deflation

During the RVOS session, the participant will lie supine in a semi-recumbent position with the lower limbs extended. A pressure cuff (SC12LTM segmental pressure cuff; Hokanson, Bellvue, WA, USA) will be applied around the proximal right thigh with a bandage wrapped underneath to avoid skin irritation and bruising. Each RVOS session will last for 40 min and will include four cycles of 5 min cuff inflation to 50 mmHg above the average systolic blood pressure to completely occlude arterial flow distally to the limb [98], followed by 5 min of complete deflation (0 mmHg) (Fig. 3). Pressure in the pneumatic cuff will be controlled by an inflator and air source device (E20 Rapid Cuff Inflator and AG101 Cuff Inflator Air Source; Hokanson). Maximum cuff pressure will be 200 mmHg.

Vital signs such as blood pressure, heart rate and the saturation of peripheral arterial blood with oxygen will be monitored before and during RVOS, and the session will be terminated if the defined criteria are met (Table 2).

**Table 2:** Termination criteria for repetitive vascular occlusion stimulus (RVOS) session

1. Heart rate < 40 bpm or > 180 bpm 2. Systolic blood pressure < 80 mmHg or > 200 mmHg 3. Mean arterial blood pressure < 60 mmHg or > 120 mmHg 4. Peripheral capillary oxygen saturation (SpO_2_) < 88% 5. Pain score > 8/10 on visual analogue scale and unwillingness to proceed with RVOS intervention 6. Objective signs of tissue injury felt due to cuff inflation	

A clinical bedside nurse will be present throughout the RVOS session and will monitor facial expression, limb movement and ventilation compliance for any sign of distress in the sedated participants. In the event that the patient experiences significant discomfort, the cuff pressure will be lowered to a pressure that is tolerated by the participant, and such adjustments will be recorded. If conscious, tolerability of the intervention will be evaluated by asking the participant to rate the pain of the procedure on a visual analogue scale (VAS). After each session, the intervention leg will be examined for signs of arterial insufficiency or tissue injury. In addition, serum creatinine kinase levels will be monitored daily as an indicator of muscle damage.

### Primary outcome measures

#### Safety

The number of serious adverse events (SAEs; DVT, pulmonary embolism and elevated serum creatine kinase concentration (creatine kinase > 3800 IU/L)) will be compared between the control and intervention groups.

#### Tolerability

The VAS pain score will be used to assess the tolerability of RVOS.

### Secondary outcome measures

#### Feasibility

Table 3 presents the pre-specified feasibility criteria that will be assessed.

**Table 3:** Feasibility criteria of the study

Trial process	Outcome measure	Feasibility criteria
Screening	Percentage of potentially eligible patients being missed	< 55% of potentially eligible patients being missed
Consent	Percentage of personal consultee/participants agreeing to enrolment	> 75% of personal consultees/participants agreeing to enrolment
Recruitment rate	Number of patients recruited	Recruit 30 patients within 16 months
Randomisation	Demographic and severity of illness in the intervention and control arms	Balanced demographic and severity of illness in intervention and control arm participants
Delivery of intervention	Percentage of RVOS sessions performed out of the total possible sessions	80% of the scheduled RVOS sessions performed
Retention rate	Percentage of patients that remain on the ICU for the full 10 days of study enrolment	> 50% of enrolled patients remain on the ICU for the full 10 days of study enrolment
Outcome measure assessments	Percentage of outcome measure assessments performed within 24 h of the scheduled time Percentage of quality-of-life questionnaires completed at 90-day follow-up	100% of RFCSA ultrasound measurements performed within 24 h of the scheduled time > 75% of vascular, strength and functional capacity measures performed within 24 h of the scheduled time > 75% of surviving patients complete the quality-of-life questionnaires at 90-day follow-up
Electronic case report form data collection	Percentage of missing outcome and clinical data	< 10% missing outcome data including ICU and hospital length of stay and survival < 10% missing clinical data obtained from clinical medical notes and electronic patient records, such as severity of illness scores and requirement for organ supportive therapies

*ICU* intensive care unit, *RFCSA* rectus femoris cross sectional area, *RVOS* repetitive vascular occlusion stimulus

#### Acceptability

A semi-structured interview conducted with the participant, personal consultee and clinical staff will be used to determine the acceptability of trial experience and RVOS intervention. Questions will relate to key trial parameters such as experience of recruitment and randomisation and participant/staff burden (see Additional file 2 for acceptability questions).

#### Muscle mass and quality

To assess muscle mass, the rectus femoris (RF) muscle of both legs (in intervention participants) and only the right leg (control participants) will be imaged using B-mode ultrasound as previously described [21, 99] at baseline and on days 6 and 11 of study enrolment. Briefly, the participant will lie in a semi-recumbent position and the RF muscle will be scanned in the longitudinal plane on the anterior aspect of the thigh, two-thirds of the distance from the anterior superior iliac spine to the superior patella border. RF ultrasound images will be analysed using ImageJ software (NIH, Bethesda, MD, USA) by blinded investigators. Muscle mass will be quantified by assessing the RFCSA, and echogenicity will be measured to evaluate the changes in muscle quality as reported previously [100, 101]. Change over time in these measures will be compared between intervention and control participants, and changes in the treated leg and the untreated leg compared in those receiving the RVOS intervention.

#### Arterial vascular measures

Arterial vascular function will be assessed at the same time points as muscle evaluation, using techniques previously described [102]. The diameter and blood flow velocity of the right superficial femoral artery at rest and immediately following 5 min of ischaemia will be measured using B-mode and pulse Doppler ultrasound imaging, respectively. Ischaemia will be induced by application of a pneumatic cuff to the right thigh and inflation to 200 mmHg cuff pressure. Blinded investigators will analyse the diameter and blood flow velocity using the commercially available software Brachial Analyzer (Vascular Research Tools 5; Medical Imaging Applications, LLC, Coralville, IA, USA) to assess flow-mediated dilation (FMD) and reactive hyperaemic response, surrogate markers of vascular health. The reactive hyperaemic response is a transient increase in blood flow in response to ischaemic stimulus, and a marker of vasodilator capacity of resistance vessels. FMD assesses endothelial-dependent vasodilation of the conduit artery in response to increased blood flow and internal wall shear stress. The resting SFA diameter and blood velocity, reactive hyperaemic response and FMD will be compared between intervention and control participants at each time point.

#### Muscle strength

On days 6 and 11 and at ICU and hospital discharge, the grip strength of the dominant hand will be assessed using a handgrip dynamometer (Takei Scientific Instruments Co. Ltd, Niigata City, Japan) and manual muscle strength evaluation of the bilateral six upper and lower limb muscle groups (shoulder abductors, elbow flexors, wrist extensors, hip flexors, knee extensors and foot dorsiflexors) performed using the Medical Research Council Sum Score (MRC-SS) [103]. Strength assessments will only be performed if the participant is sufficiently alert and free from delirium.

#### Physical function

Mobility will be assessed in the ICU on days 1, 6 and 11 of study enrolment (or at ICU discharge, whichever is earlier) and at hospital discharge, using the ICU Mobility Scale, a validated measure with excellent clinometric properties [104, 105]. In addition, functional assessment (‘timed up and go’ and ‘sit to stand’) will be performed at hospital discharge. ‘Timed up and go’ will involve measuring the time taken to stand up from a standard chair, walk a 3-m distance at normal pace and return to the chair; for ‘sit to stand’, the number of times that the participant is able to completely stand upright from a standard chair and sit back down fully within 30 s will be assessed. Due to the study population being frail, use of aids will be permitted and documented.

#### Clinical outcomes

Clinical outcomes between participants in the intervention and control arms will be compared: incidence and duration of delirium; incidence, duration and severity of acute kidney injury (assessed using the AKIN classification) [106]; duration of specific organ support (mechanical ventilation, renal replacement therapy, mechanical or pharmacological cardiovascular support); days alive and without a ventilator in the first 28 days of hospital stay; length of ICU and hospital stay; and hospital and 3-month mortality.

#### Laboratory assessment

A 10-ml urine sample (collected from an indwelling urinary catheter) and 1 × 10 ml EDTA, 3 × 4 ml EDTA, 1 × 4 ml serum, 1 × 3 ml Tempus™ RNA blood tube samples will be collected on days 1, 6 and 11 of study enrolment from indwelling arterial cannulae, and via venepuncture at hospital discharge. In addition, 1 × 4 ml EDTA blood samples will be taken at 3 and 24 h after the first RVOS session in patients randomised to the intervention arm and similarly at 3 and 24 h after baseline assessment in control participants. Plasma and serum will be separated from the EDTA and serum tubes, and the urine sample and Tempus™ blood RNA will be stored at − 80 °C. The, 1 × 10 ml EDTA will be processed to isolate peripheral blood mononuclear cells (PBMCs) using the Optiprep™ protocol [107], which will be stored at − 80 °C until analysis.

Samples will be stored in accordance with the Human Tissue Act (2004) beyond the end of the trial to allow for various laboratory analyses to evaluate mechanisms behind the effect of RVOS. Biomarkers that will be analysed are presented in Table 4. Further biomarkers may be studied to evaluate mechanisms of effect as scientific evidence dictates.

**Table 4:** Laboratory analysis

Sample	Biomarkers
Plasma and serum	Muscle anabolic marker insulin-like growth factor 1 Muscle catabolic marker Myostatin Inflammatory cytokines interleukin (IL)-6, IL-10, tumour necrosis factor α, granulocyte–macrophage colony-stimulating factor, macrophage inflammatory protein 1, transforming growth factor β1 Angiogenic factors vascular endothelial growth factor, hypoxia inducible factor 1α
Tempus RNA tube	MicroRNAs linked with muscle atrophy such as miR-29b, miR-542-5p and miR-424-5p [108]
Peripheral blood mononuclear cells	DNA damage/repair enzymes GAD45a and APE-1
Urine	Oxidative stress-derived DNA damage marker 8-hydroxy deoxyguanosine

### Statistical analysis

The statistical analysis addresses the objectives of demonstrating the safety and tolerability of applying RVOS to the lower proximal limb of ICU patients, and assessing the feasibility of undertaking a larger-scale study to demonstrate efficacy. As a feasibility trial, the statistical analysis will primarily be exploratory in nature, and thus hypothesis generating rather than confirmatory.

The primary objective of safety will be evaluated based on the frequencies and percentages of adverse events categorised by severity, group and site. The tolerability will be evaluated by assessing VAS pain scores of the intervention arm at each time point in terms of means and standard deviations.

The main feasibility outcome of recruitment will be evaluated as the percentage of randomised participants out of total eligible patients. The number of participants randomised as a percentage of targeted recruitment will also be computed. If data are available, the average number of research assistants’ hours spent on recruiting one participant will also be computed. Retention will be evaluated as the proportion of participants remaining at each time point compared to the number recruited in each arm.

Descriptive statistics will be used to summarise all response variables by site, time point and treatment group. Means and standard deviations will be computed for continuous outcomes and frequencies and percentages will be computed for categorical/dichotomous outcomes. The relationship with potential confounding factors—such as site, demographic data, delirium during ICU stay, severity of illness, length of ICU stay, sepsis (defined using SOFA score), and use of drug treatment (such as corticosteroids, neuromuscular blocking drugs, statins and propofol)—will be explored.

The following inferential statistics will be performed strictly for the purpose of identifying the directions and magnitudes of effects rather than to evaluate the statistical significance of the results due to inadequacy of statistical power.

Outcome measures recorded at a time point (such as ‘timed up and go’ and ‘sit to stand’ assessment) and clinical outcomes (such as duration of mechanical ventilation and length of ICU and hospital stay) will be compared between the two arms using an independent-sample *t* test.

Repeated outcome measures such as the RFCSA and echogenicity, superficial femoral artery measures (resting diameter and blood flow, FMD and reactive hyperaemic response), ICU mobility score, MRC-SS and handgrip strength data will be analysed separately as the response variable in a general linear mixed model using SAS PROC MIXED, with group (the dichotomy of standard care or RVOS), site, baseline measure, time and group × time interaction as fixed effects and subject as random effects. Appropriately selected covariates will be included in the model as fixed effects to adjust for the variability caused by these covariates.

All statistical analyses will be performed by the Trial Statistician at Surrey Clinical Trials Unit (CTU) according to a pre-specified Statistical Analysis Plan, which will be finalised and signed off before the database lock.

### Data collection and monitoring

All study data will be collected in compliance with the principles of Good Clinical Practice (GCP) and with the Data Protection Act. Patients’ medical notes will be used as source documents for clinical data, while data related to the RVOS intervention (e.g. time of session, cuff pressure, VAS score) and outcome measure assessments will be recorded in a paper workbook before transcribing to a secure web-based electronic data capture system (Promasys) provided by Surrey CTU. The Surrey CTU data management team and sponsor (RSCH) will jointly oversee the administration of study documentation to ensure that study data are authentic, accurate and complete. The sponsor’s Quality and Assurance Officer will conduct monitoring visits to review the source documentation and electronic case report forms (eCRF) and to evaluate them for accuracy, completeness and compliance with the approved protocol, applicable regulations and GCP standards. The frequency of the monitoring visits will depend on the findings of the first patient-monitoring visit at each site.

Participants’ anonymity will be maintained at all times, and study workbooks and other documents will only be labelled with a non-identifiable study participant number. Paper documents that identify participants (such as consent forms) will be stored in a separate folder in a locked office of the secured department site and maintained by investigators in strict confidence. At the end of the trial, any non-essential confidential documents will be destroyed and essential documents will be kept securely for at least 10 years after study completion.

### Trial monitoring groups

Day-to-day management of the trial will be the responsibility of the Trial Management Group (TMG), members of which will include the Chief Investigator and co-investigators, the research assistant, the CTU Trial Statistician and Data Manager, and the sponsor’s Quality and Assurance Officer. The group will monitor all aspects of the conduct and progress of the trial including recruitment figures, data quality and protocol adherence. In addition, experts in the study research field (consultant intensivists, Chief and Principle Investigators, sponsor and CTU representatives) and patient and public involvement (PPI) members have been appointed as members of a Trial Steering Committee (TSC). The TSC will be responsible for overseeing conduct of the trial and will convene every 6 months, will review trial progress, recruitment rates, safety data (reviewed blinded to treatment allocation) and ethical issues, and will make recommendations for protocol modifications. In the case of major safety concerns, the TSC will be able to request unblinded review of the safety data.

In order to safeguard the interests of trial participants, an independent Trial Safety Group (TSG) has been appointed and will be updated with safety data after every five patients recruited or in the event of an SAE occurring. The TSG will be responsible for monitoring any evidence for treatment harm, informing the TSC of any safety concerns and advising with regard to protocol modifications suggested by investigators or sponsors.

Four previous ICU-AW sufferers and their carers who constitute our Project Advisory Group (PAG) were involved in defining the research questions and in aiding development of a patient-friendly study design. The PAG has reviewed and advised on language and content of participant-related study documents. Some members are also TSC members and will be involved in the delivery and management of the trial. At the end of the study, they will also assist in writing a letter thanking participants for their involvement and lay summary of findings.

### Serious adverse events

Safety reporting will be performed in accordance with HRA guidelines for non-CTIMP research (not involving investigational medicinal products or medical devices) with adherence to GCP standards. Untoward medical occurrences are expected in critically ill patients and their monitoring and treatment is considered standard care. As a result, only serious adverse events (SAEs) will be reported. These are defined as any untoward occurrence that results in death; is life-threatening; requires hospitalisation or prolongs existing hospitalisation; or results in persistent or significant disability/incapacity; or any other important medical event that required intervention to prevent the outcomes already listed based upon appropriate judgement by the investigators.

SAEs will be recorded from when participant consent is obtained to 3-month follow-up after hospital discharge. SAEs possibly, probably and definitely related to the study, such as DVT, pulmonary embolism and elevated creatine kinase (creatine kinase > 3800 IU/L), will be recorded. For all SAEs, the following data will be recorded in the eCRF and patient notes: description; date and time of occurrence; severity; relationship of SAE to study procedure (determined by the investigator physician); treatment required; and action taken with research procedure. The completed SAE form, signed by the reporting investigator and Principle Investigator of the site, will be submitted to the sponsor within 24 h of becoming aware of the event and all SAEs will be followed-up until resolved. If unexpected serious related event occurs, the Chief Investigator will notify Principle Investigators at local sites and the research ethics committee within 15 days of the event.

In the case of unanticipated concerns of safety to study participants or availability of new data arising from clinical or preclinical studies with this intervention, the study will be paused during review of newly available data prior to a final decision for continuation or termination of the study. If, in the opinion of the Chief Investigator, the clinical events indicate that it is not justifiable to continue the trial, the TSC will terminate the trial following consultation with the sponsor.

### Indemnity

The Royal Surrey County Hospital Foundation Trust, as the trial sponsor, holds professional liability insurance to meet the potential legal liability of the sponsor and employees for harm to participants arising from the design and management of the research.

Indemnity to meet the potential legal liability of the investigators/collaborators for the harm of participants arising from the conduct of the research is provided by the NHS Indemnity scheme or through professional indemnity.

### Dissemination of results

Study results will be published in peer-reviewed journals and presented at international/national surgical, anaesthetic and perioperative medicine conferences.

In addition, a lay summary of findings will be disseminated to study participants.

## Discussion

This study will be the first to assess the safety, tolerability, feasibility and acceptability of RVOS in ICU patients. The effect of RVOS on muscle mass atrophy and weakness, and on arterial dysfunction observed in ICU patients, will also be explored.

Thirty-two ventilated critically ill patients with at least two other organ failures will be recruited to the study. Multiple organ failure patients were chosen as the study population because these patients undergo more pronounced muscle wasting (16% decrease in the RFCSA compared to 3% in single organ failure patients within 7 days of ICU admission) [21].

RVOS will be performed using equipment conventionally and safely used for vascular disease diagnostics and routinely used during vascular and orthopaedic operations. Control participants will receive standard care and no sham treatment (repeated cuff inflation at lower pressure) so as to avoid any detrimental impacts of isolated venous occlusion. Participants will thus not be blinded in the study. However, this is unlikely to introduce bias to the objective measures as muscle and arterial ultrasound images will be anonymised and analysed blinded to subject identity, study time points and group allocation.

The tolerability of RVOS cannot be assessed when participants are ventilated and sedated. Even when participants are extubated and off sedation, delirium (an acute brain dysfunction) is common in critically ill patients and can influence the VAS score. CAM-ICU assessment will be performed and the mental state of the participant during the RVOS session will be recorded to observe any effects of delirium on the VAS score.

Ultrasound assessments of muscle mass and quality and arterial function can be performed when participants are sedated; however, muscle strength and physical function measures are volitional assessments and require the participant’s full attention and cooperation. Therefore, sedation, lack of willingness to participate or lack of mental capacity due to delirium can prevent these outcome assessments. These measures will therefore only be performed when the participant is off sedation and CAM-ICU negative; this could lead to the time points when the strength and physical function assessments are performed varying between participants.

Our study population has a high risk of hospital death and post-discharge mortality. This means that participants could be lost due to death before completion of the study, with previous reports of 30% mortality in a study with similar inclusion and exclusion criteria [109]. We will recruit until we have 32 participants that survive the ICU stay, recognising that subsequent deaths after ICU discharge may reduce the data available at hospital discharge and at 3-month follow-up.

We expect repeated inflation and deflation of the pneumatic cuff during the RVOS to cause minor fluctuations in heat rate and blood pressure. As a result, patients with profound cardiovascular instability (defined by infused vasopressors ≥ 0.5 μg/kg/min of norepinephrine or by opinion of the senior attending physician) will not be studied. Moreover, during the RVOS session, haemodynamic parameters will be monitored closely. Critically ill patients have a 10% incidence rate of DVT [110], whilst an incidence of 0.055% is reported in others who have received blood flow restriction exercise [92]. We will thus exclude patients with a history of DVT and pulmonary embolism (PE) as well as patients who have contraindication to pharmacological venous thromboembolism prophylaxis. Apart from an incidence of minor events such as skin petechiae (4.4%) and subcutaneous bruising (13.1%), no major bleeding risk of RVOS has been reported in the literature [92, 111]. However, as a safety measure, patients with profound coagulopathy (defined by > 2.5 times normal prothrombin time, > 2 times normal APTT, platelet count ≤ 50 or on IV heparin infusion APTR ≥ 2) will not be recruited or studied if this occurs.

If our pilot study shows that RVOS is safe, feasible and acceptable to apply to ICU patients, an appropriately-powered randomised controlled trial will be designed to assess any potential benefit of this procedure.

## Trial status

The study was opened to recruitment at the RSCH site in October 2017 and at the ASPH site in January 2018, and the first participant was recruited in January 2018. Study recruitment was completed in Feburary 2019.

## Additional files

Additional file 1: Standard Protocol Items: Recommendations for Interventional Trials (SPIRIT) 2013 checklist: recommended items to address in a clinical trial protocol and related documents (DOC 137 kb)

Additional file 2: Acceptability questions (DOCX 14 kb)

## Data Availability

The data generated from this study will be made available on request to the corresponding author.

## Abbreviations

APTR: Activated partial thromboplastin time ratio
APTT: Activated partial thromboplastin time
CAM-ICU: Confusion assessment method for the ICU
CPAP: Continuous positive airway pressure
DVT: Deep vein thrombosis
FMD: Flow-mediated dilation
GCP: Good Clinical Practice
ICU: Intensive care unit
ICU-AW: Intensive care unit-acquired weakness
MRC-SS: Medical Research Council Sum Score
NIV: Non-invasive ventilation
PAG: Project advisory group
PBMC: Peripheral blood mononuclear cell
RFCSA: Rectus femoris cross-sectional area
RVOS: Repetitive vascular occlusion stimulus
SAE: Serious adverse event
SFA: Superficial femoral artery
SOFA: Sequential Organ Failure Assessment
TMG: Trial Management Group
TSC: Trial Steering Committee
TSG: Trial Safety Group
VAS: Visual analogue scale
VEGF: Vascular endothelial growth factor
