# Supplementary material for: Repetitive vascular occlusion stimulus (RVOS) versus standard care to prevent muscle wasting in critically ill patients (ROSProx):a study protocol for a pilot randomised controlled trial
Source: Trials. 2019 Jul 24;20:456. doi: 10.1186/s13063-019-3547-5 (PMC6657179; doi:10.1186/s13063-019-3547-5)
Supplement: Supplementary file 2 — Acceptability questions (DOCX 14 kb) [file 13063_2019_3547_MOESM2_ESM.docx]

**Acceptability Questions**

| **Control arm participant questions** |
| --- |
| 1. How did you find being in the study? |
| 1. What could we have done to make it better for you? |
| 1. How could we improve the paperwork (e.g. consent form etc.)? |
| **Intervention arm participant questions** |
| 1. How did you find being in the study? |
| 1. What could we have done to make it better for you? |
| 1. How could we improve the paperwork (e.g. consent form etc.)? |
| 1. Have you noticed any difference between your legs? |
| 1. How did you find the intervention? |
| 1. If the intervention was found to be effective at reducing muscle weakness, it is an acceptable treatment.   Strongly Agree  Agree  Neutral  Disagree  Strongly Disagree |
| **Personal consultee/ Staff questions** |
| 1. How did you find being the personal consultee/ having your patient in the study? |
| 1. What could we have done to make it better for you? |
| 1. How could we improve the paperwork (e.g. consent form etc.)? |
| 1. What did you think of the intervention? |
